# Application of Surface Electromyography in Exercise Fatigue: A Review

**DOI:** 10.3389/fnsys.2022.893275

**Published:** 2022-08-11

**Authors:** Jiaqi Sun, Guangda Liu, Yubing Sun, Kai Lin, Zijian Zhou, Jing Cai

**Affiliations:** College of Instrumentation and Electrical Engineering, Jilin University, Changchun, China

**Keywords:** exercise fatigue, sEMG, machine learning, feature extraction, classification

## Abstract

Exercise fatigue is a common physiological phenomenon in human activities. The occurrence of exercise fatigue can reduce human power output and exercise performance, and increased the risk of sports injuries. As physiological signals that are closely related to human activities, surface electromyography (sEMG) signals have been widely used in exercise fatigue assessment. Great advances have been made in the measurement and interpretation of electromyographic signals recorded on surfaces. It is a practical way to assess exercise fatigue with the use of electromyographic features. With the development of machine learning, the application of sEMG signals in human evaluation has been developed. In this article, we focused on sEMG signal processing, feature extraction, and classification in exercise fatigue. sEMG based multisource information fusion for exercise fatigue was also introduced. Finally, the development trend of exercise fatigue detection is prospected.

## Introduction

Exercise fatigue is a physiological phenomenon that originates from human activities and results in decreased physical performance (Nijs et al., 2011). The main manifestation of exercise fatigue is muscle fatigue which was defined as the “failure to maintain the force output, leading to a reduced performance” (Asmussen, 1993). Muscle strength declines progressively during exercise, so fatigue occurs before the task failure (Gandevia, 2001). There are many examples of application fields involving muscle fatigue, including sports (Simsek, 2017; Kuniszyk-Jozkowiak et al., 2018; Liu et al., 2019; Hedayatpour et al., 2021; Liu and Li, 2021; Rodriguez-Rosell et al., 2021), rehabilitation (Gaudet et al., 2018; Kim et al., 2018; Meng et al., 2019; Na et al., 2020; Fundaro et al., 2021; Sato et al., 2021), and occupation (Alberto et al., 2018; Fang et al., 2021; Ji and Huang, 2021; Yu et al., 2021; Gonzalez-Zamora et al., 2022). Physiological signals, biochemical assessments, and questionnaires are used to monitor exercise fatigue (Halson and Shona, 2014). Questionnaires are subjective. Invasive biochemical tests cause discomfort to the subjects. Noninvasive physiological testing with scientific and statistical approaches can provide confidence and certainty.

Muscle fatigue is divided into central and peripheral components. Peripheral fatigue is caused by changes in the neuromuscular junction. Central fatigue originates in the central nervous system (CNS). The production of skeletal muscle force depends on contractile mechanisms, and failure of nervous, ion, vascular, and energy systems can contribute to the development of muscle fatigue (Wan et al., 2017). During sustained contractions, metabolic changes in the muscle affect the propagation of action potential. These changes result in a progressive reduction of muscle fiber conduction velocity (CV). This is one of the main causes of the changes in amplitude and spectral EMG variables during fatigue. This physiological variable provides a relevant means to describe and quantify the muscle fatigue (Marco et al., 2017).

Exercise fatigue can be detected through surface electromyography (sEMG) (Chang et al., 2012). Constant exercise load results in the rise of EMG activity during fatigue (Chlif et al., 2018). Electrical currents generated by muscle contractions can be monitored in the form of EMG signals and displayed on a computer (Barszap et al., 2016). Bioelectricity is detectable when muscle contracts (Tang et al., 2020). Electromyographic techniques are used in sports and rehabilitation accepted by researchers (Cortez et al., 2017; Pakosz and Konieczny, 2020; Quittmann et al., 2020). EMG can be recorded by needles inserted into the muscle (Kwon et al., 2018) or electrodes placed over the skin surface (Zeng et al., 2021). Compared with invasive EMG, sEMG is more popular among researchers (Khan et al., 2019; Yamagishi et al., 2019; Silva et al., 2020).

This review offers an overall summary of the assessment of human exercise fatigue based on sEMG signals and highlights signal processing, feature extraction, and classification. The possibilities for future work on exercise fatigue with sEMG will also be discussed in this study.

## Acquisition of Surface Electromyography and Preprocessing

The sEMG is a biological electric signal generated by muscle contraction that can be harvested by electrodes. The sEMG signals are the most intuitive physiological signals of muscle activity and the best means to detect muscle fatigue. The sEMG signal is a kind of pseudorandom physiological signal that is very weak. The voltage of the sEMG signal range from 50 μV to 100 mV and the frequency is varied from 10 to 500 Hz (Pancholi and Joshi, 2018). The electrode skin impedance which is one of the noises that affects the quality of EMG signals must be as low as possible to obtain effective signals (Sae-Lim et al., 2019). Pancholi and Joshi (2018) obtained sEMG signals from five different arm muscles using hardware based on ADS1298 IC (Texas Instruments) and ARM cortex M4 series processor with 4,000 Hz sampling frequency. De la Pena et al. (2019) recorded sEMG-related muscle fatigue in sports training using a portable prototype with a 5,000 Hz sampling rate. Zhao et al. (2020) acquired sEMG data based on a software platform for visualizing sEMG information on muscle fatigue during upper limb rehabilitation training. In Makaram et al. (2021), Biopac MP 36 (Biopac Systems Inc. CA, United States) was used to acquire brachii muscle of 52 healthy participants’ sEMG during dynamic contractions at an acquisition rate of 10,000 Hz. Wang L. et al. (2021) used wearable sampling electrodes that the sampling frequency is 200 Hz to collect sEMG signals in real-time during the driving tasks. Chen et al. (2021b) used the MP160 physiological record analysis system produced by the American company BIOPAC to analyze the fatigue of miners.

Exact electrode positioning is vital for obtaining reliable EMG signals. A study showed high correlations between all electrode sites and clavicular movements. The traditional electrode site record more informative signals in subjects (Zanca et al., 2014). All trunk muscles were affected by electrode position changes, but the abdominal muscles were more affected than the back muscles (Huebner et al., 2015). Ghapanchizadeh et al. (2016) found that the optimal signal from the flexor carpi radialis muscles was presented at 90% and for the extensor carpi radialis muscles was shown at 90% of the electrode position over the forearm length. The muscle moves away from its uncontracted position directly under the EMG electrode when contracts. Elsais et al. (2020) used ultrasound to track the relative motion between skin and muscle to quantify the magnitude of the movement between them and inform protocols for surface EMG placement.

The raw sEMG data collected will inevitably be mixed with power line interference and motion artifacts (Zheng and Hu, 2019). Therefore, effective preprocessing is required before feature extraction of these signals (Ahmadizadeh et al., 2021). Common methods of sEMG preprocessing include filtering (Zhao et al., 2020), normalization, and windowing (Fang et al., 2020). Tapia et al. (2017) used independent component analysis (ICA) and empirical mode decomposition (EMD) to process sEMG signals. (Wu et al., 2017) achieved better accuracy for the diagnosis of muscular fatigue through ensemble empirical mode decomposition (EEMD) by Hilbert transform (HT). Zhang et al. (2019) decomposed sEMG signals by principal component analysis (PCA) into principal components and weight vectors that improve the validity of parameters. In Avian et al. (2022), Discrete Wavelet Transform (DWT) is used to process the sEMG signal to increase model performance.

The increase from baseline represents the onset of muscle activity. Muscle activity onset can be estimated from EMG and ultrasound (Dieterich et al., 2017). Gupta et al. (2014) determined the onset of medial gastrocnemius muscle activity using visual and automated methods during a stretch-shorten-cycle muscle action. Zhang and Zhou (2012) proposed a novel method of muscle activity onset detection based primarily on the sample entropy (SampEn) analysis of the surface EMG. Liu et al. (2015) presented an unsupervised EMG learning framework based on a sequential Gaussian mixture model (GMM) to detect muscle activity onsets. Appropriate signal preprocessing method is helpful to improve the effectiveness of feature extraction.

## Feature Extraction

Feature extraction from the sEMG signal plays an important role in the accuracy of fatigue detection. Time domain, frequency domain, time-frequency domain, and nonlinear parameters are four major types in sEMG-based signal processing (Too et al., 2018b; Yousif et al., 2019; Bukhari et al., 2020).

## Time-Domain Feature Analysis

The calculation of time-domain features is the most popular approach for sEMG feature extraction that is directly calculated with time as the independent variable. In the time domain, the sEMG signal is usually regarded as a random signal whose mean value is zero and variance varies with signal intensity. As the calculation of time-domain features is simple and intuitive, it is a widely used feature extraction method of sEMG signal in human movement (Harmon et al., 2021). The typical time-domain features of sEMG signals mainly include the root mean square (RMS) (Cui et al., 2021), integrated EMG (iEMG) (Alam et al., 2020), zero-crossing rate (ZCR) (Kim et al., 2018), waveform length (WL), variance of electromyography (VAR) (Whittaker et al., 2019), and mean absolute value (MAV) (Chapman et al., 2019).

With the occurrence of muscle fatigue, the time domain features of sEMG generally show an upward trend over time (Goubault et al., 2022). RMS and iEMG not only reflect the amplitude changes of the sEMG signal in the time domain but also clearly reflect the biomechanical properties and muscle energy changes in the exercise process (Silvetti et al., 2017; Wu et al., 2017). Therefore, RMS and iEMG are often used to indicate muscle activation intensity and human motion state (Triwiyanto et al., 2018).

## Frequency-Domain Feature Analysis

The frequency-domain features are spectrum or power spectrum (PS) features that are obtained by fast Fourier transform (FFT) to the original sEMG signals, thus the frequency band distribution of the signals can be observed directly (Chandra et al., 2020). Researchers believe that frequency domain analysis is more meaningful than time domain analysis in both static and dynamic motion. To quantitatively describe the spectrum and PS features of sEMG signals, the mean frequency (MF) (Hou et al., 2021) and median frequency (MDF) (Park and Park, 2021) are generally used that decrease linearly over time. The deeper the muscle fatigue, the faster the MF decreases. Both MF and MDF can represent the frequency of measured muscle CV, but in practical application, MDF is more sensitive than MF in reflecting muscle activity and functional state. MF can also get good results in muscle fatigue detection (Chai et al., 2019). Significant changes in the PS indicate muscle fatigue, and the PS drifts from high frequency to low frequency. For example, after maximum weight training, the peak value of the sEMG signal will increase and drift to the lower frequency band. During extreme contraction, there is a decrease in muscle fiber action potential and possible emission frequency.

## Time-Frequency Distributions

To make up for the deficiency of Fourier transform analysis of non-stationary signals, time-frequency features of sEMG signals are introduced. The analysis of time-frequency features is important to the estimation of muscle fatigue state, and it is usually necessary to analyze both the time-domain and frequency-domain features of sEMG signals to obtain comprehensive information of muscle physiological changes. Traditional sEMG time domain and frequency domain analysis methods only describe the time domain or frequency domain features, but the time-frequency domain feature analysis method can overcome this limitation. At present, the available time-frequency analysis methods for sEMG signal mainly include Short-time Fourier Transform (STFT), Wavelet Transform (WT), Choi William distribution (CWD), and Wigner-Ville distribution (WVD), used for visual observation of signal frequency content evolution over time. WVD is a common bilinear time-frequency distribution, which is the most common type of Cohen’s time-frequency distribution. Instantaneous frequency parameters commonly used are instantaneous mean frequency (IMNF) (Triwiyanto et al., 2017) and instantaneous median frequency (IMDF) (Yousif et al., 2019), which show a downward trend with the deepening of fatigue degree. Average instantaneous MF has higher stability and sensitivity than frequency-domain features. Yousif et al. (2019) applied IMNF and IMDF to assess the muscles fatigue of the male runner during 400 m running with three types of running strategies.

## Non-Linear Parameters

The sEMG complexity and entropy decrease linearly with the increase of fatigue degree. The complexity of Lempel-Ziv [C (n)] (Jo et al., 2018) is the speed at which new patterns appear with the increase of the length of time series, indicating the degree of randomness of the series, which decreases linearly in the process of dynamic motion. In dynamic motion, approximate entropy rises first and then decreases for most people. Marginal spectrum entropy (MSE) (Jero and Ramakrishnan, 2019) is a useful real-time muscle fatigue assessment method with the advantages of fast, reliable assessment of muscle fatigue and anti-noise. Compared with approximate entropy and MDF, MSE can be calculated quickly, the data length robustness is better, and muscle fatigue can be assessed reliably. It has high stability for different individuals and good noise resistance. SampEn (Cui et al., 2017), proposed by Richman and Moorman in 2000, measures the probability of generating new patterns in signals by measuring the complexity of time series. SampEn has a strong anti-noise ability and can reduce data deviation. The higher the complexity of the sequence, the higher the entropy. The multi-scale entropy (Fan et al., 2018) of sEMG signals decreases as the load increases, which more accurately represents the complexity of the muscle system. Compared with the traditional SampEn, it can more objectively reflect the working status and fatigue grade of the muscle. Multi-scale entropy analysis has a small amount of calculation and can adapt to the complexity of dynamic muscle contraction under time-varying loads. In practical work, normalized average multi-scale entropy can be used as a quantitative index to measure the dynamic fatigue degree of muscles. Recurrence quantification analysis (RQA) (Chen et al., 2018) is used to determine the percentage of line segments (%DET) reflecting the periodicity of signals. The RQA software is used to calculate and determine the percentage of line segments. Under dynamic and static loads, %DET increases linearly with the occurrence of muscle fatigue. Lejun et al. (2018) used C(n) to evaluate fatigue in all-out cycling exercises. Cui et al. (2017) used SampEn to investigate the fatiguing features of muscle-tendon units (MTUs) within skeletal muscles during static isometric contraction tasks. Hernandez and Camic (2019) investigated the effect of fatigue status and contraction type on the complexity of the sEMG signal, using SampEn and Detrended Fluctuation Analysis (DFA). Kahl and Hofmann (2016) compared the performance of different fatigue detection algorithms quantifying muscle fatigue based on sEMG signals. Fatigue detection algorithms including spectral moments ratio (SMR), SampEn, fuzzy approximate entropy (fApEn), and RQA (%DET) were calculated. After identifying the extracted features from the sEMG signal, the next important step is classification to detect the fatigue state.

## Classification

With the development of machine learning, machine learning algorithms were widely applied to exercise fatigue classification. Classification in this article normally refers to supervised learning where individuals are classified based on their features. Some classification algorithms based on sEMG are listed in Table 1, mainly including fuzzy logic (FL) (Li, 2017), hidden Markov model (HMM) (Shahmoradi et al., 2017), k-nearest neighbor (KNN) (Bukhari et al., 2020), support vector machine (SVM) (Chen et al., 2021b), linear discriminant analysis (LDA) (Ahmed et al., 2020), and artificial neural network (ANN) (Subasi and Kiymik, 2010).

**TABLE 1:** Machine learning algorithms for fatigue classification.

References	Subjects	Channels	Location	Model	Accuracy (%)
Shahmoradi et al., 2017	6	1	Anterior deltoid muscle	HMM	95.3
Chen et al., 2021b	40	1	Brachioradialis of the forearm	RF	90
Wang et al., 2020	20	5	Lower limbs	CNN-SVM	86.69
Subasi and Kiymik, 2010	14	1	Biceps brachii	ANN	90
Bharathi et al., 2022	58	1	Biceps brachii	ELM	94.09
Zhang et al., 2021	10	1	Upper limb	MFFNet	77.37
Wang J. H. et al., 2021	20	4	Lower limbs	LSTM	95.18
Rejith et al. (2016)	30	1	Bicep muscle	MLPNN	60.12
Chen et al., 2021a	55	1	Brachioradialis of the forearm	XG-Boost	89.47

Support vector machine is a popular machine learning classification method because it is simple, fast, and stable, and shows better accuracy than other methods (Karthick et al., 2018; Wang et al., 2018; Greco et al., 2019; Wang, 2021). Since the features extracted from the sEMG signal are the input of the classifier, the accuracy of classification results is closely related to the feature extraction method. Dimension reduction plays a crucial role in index extraction, which can reduce the calculation time. Common dimension reduction methods include PCA (Qi et al., 2020) and ICA (Too et al., 2018a).

Khan et al. (2019) used the random forest trained by distributive power frequency of the sEMG signal of the vastus-lateralis muscle to predict muscle fatigue. They obtained a high accuracy in fatigue classification. Wang et al. (2020) proposed a muscle fatigue classification method based on sEMG signals to detect muscle fatigue by the Convolutional Neural Network–Support Vector Machine (CNN-SVM), Support Vector Machine, Convolutional Neural Network, and Particle Swarm Optimization–Support Vector Machine algorithms. CNN-SVM algorithm achieves the highest accuracy rate in muscle fatigue classification.

Zhang et al. (2021) detected muscle fatigue based on the Multidimensional Feature Fusion Network (MFFNet), which is composed of Attention Frequency domain Network (AFNet) and Attention Time-domain Network (ATNet). The result shows 77.37% higher than other classifiers. Bharathi et al. (2022) developed an automated muscle fatigue detection system and acquired 58 healthy volunteers’ signals under dynamic muscle fatiguing contractions. The extreme learning machine (ELM) model performs well with a 94.09% result. Wang J. H. et al. (2021) proposed a new muscle fatigue recognition model based on the long short-term memory (LSTM) network. Rejith et al. (2016) estimated the elbow kinematics under fatigue using sEMG by Multi-layered Perceptron Neural Network (MLPNN) which gave a classification accuracy of 60.12%. Chen et al. (2021a) studied fatigue of miners with physiological signals by extreme gradient boosting (XG-Boost).

## Surface Electromyography Based Multisource Information Fusion for Exercise Fatigue

Multi-sensor fusion based on sEMG can collect more dimensions of human activities from multiple dimensions. Thus, it is more comprehensive than fusion methods that use data from a single sensor. Qi et al. (2018) proposed a method for driving fatigue assessment based on the electroencephalogram (EEG) and EMG. Zhao et al. (2020) proposed a wearable monitoring device by integrating electrocardiogram and electromyogram (ECG/EMG) sensors to acquire data for monitoring fatigue during rehabilitation training. Martinez-Aguilar and Gutierrez (2019) analyzed cortico-muscular and cortico-cardiac coupling to study the development of muscular fatigue by electromyography (EMG), electrocardiography (ECG), and electroencephalography (EEG). Bilgin et al. (2015) presented a muscle fatigue detection method based on the frequency spectrum of EMG and mechanomyogram (MMG). Scano et al. (2020) studied the combined use of NIRS and sEMG in muscle fatigue assessment.

## Discussion and Conclusion

Fatigue detection based on surface EMG has important application value in sports training, rehabilitation treatment, and movement recognition. This article aimed to provide an overview of sEMG signal processing, feature extraction, and classification in exercise fatigue. In real-time detection, portability of the device, removal of artifacts, feature extraction, and classification techniques should be properly investigated. Using appropriate methods can remove noise to improve EMG signal quality. With the increase in the number of EMG channels and features, it is necessary to choose a reasonable dimensionality reduction method. The methods should greatly reduce the computational complexity of the classifier and preserve maximum information of the signal. Classification techniques have been extensively studied, but their timeliness and generalization still have research significance. A combination of processing methods and pattern recognition techniques may be helpful to increase the classification speed and accuracy. And the adaptability of good algorithms to fresh samples needs further study. Finally, we propose that the current review can be used as a guide for further improving exercise fatigue assessment based on sEMG for various applications of the human body.

## Author Contributions

JS mainly wrote the manuscript under the guidance of GL and JC. YS, KL, and ZZ participated in the analysis. All authors contributed to the article and approved the submitted version.

## Conflict of Interest

The authors declare that the research was conducted in the absence of any commercial or financial relationships that could be construed as a potential conflict of interest.

## Publisher’s Note

All claims expressed in this article are solely those of the authors and do not necessarily represent those of their affiliated organizations, or those of the publisher, the editors and the reviewers. Any product that may be evaluated in this article, or claim that may be made by its manufacturer, is not guaranteed or endorsed by the publisher.
